# Uptake Patterns of Untreated Primary Gastrointestinal Extranodal Lymphomas on Initial Staging ^18^F-FDG PET/CT and Metabolic Tumor Parameters

**DOI:** 10.4274/mirt.48658

**Published:** 2017-10-02

**Authors:** Engin Alagöz, Kürşat Okuyucu, Semra İnce, Murat Kantarcıoğlu, Şükrü Özaydın, Cumhur Heper, Türker Türker, Nuri Arslan

**Affiliations:** 1 Gülhane Training and Research Hospital, Clinic of Nuclear Medicine, Ankara, Turkey; 2 Gülhane Training and Research Hospital, Clinic of Gastroenterology, Ankara, Turkey; 3 Gülhane Training and Research Hospital, Clinic of Medical Oncology, Ankara, Turkey; 4 İstanbul University Institute of Cardiology, Clinic of Nuclear Medicine, İstanbul, Turkey; 5 Gülhane Training and Research Hospital, Clinic of Public Health, Ankara, Turkey

**Keywords:** ^18^F-fluorodeoxyglucose positron emission tomography/computed tomography, metabolic tumor parameters, primary gastrointestinal lymphoma

## Abstract

**Objective:**

Non-Hodgkin’s lymphomas arising from tissues other than primary lymphatic sites are classified as primary extranodal lymphomas (PEL). PELs of the gastrointestinal system (PGISL) originate from the lymphatic tissues within the gastrointestinal tract. The prognostic value of ^18^F-FDG PET/CT in lymphomas is high in terms of both overall survival (OS) and disease-free survival (DFS). Our aim was to investigate the uptake patterns and properties of low-grade and high-grade PGISL on primary staging ^18^F-FDG PET/CT, as well as the prognostic significance of metabolic tumor parameters in high grade PGISL.

**Methods:**

Thirty-nine patients with PGISL were enrolled in this retrospective cohort study between 2004-2015. Primary staging ^18^F-FDG PET/CT have been performed and quantitative parameters of SUV_max_, SUV_mean_, metabolic tumor volume (MTV), total lesion glycolysis (TLG) have been calculated for all patients prior to treatment. Low-grade and high-grade PGISL were compared in terms of metabolic tumor parameters. Cox regression models were performed to determine factors that correlate with DFS in high-grade PGISL.

**Results:**

There were statistically significant differences between high-grade and low-grade PGISL in terms of SUV_max_, SUV_mean_, MTV, TLG, recurrence, mortality, DFS and OS. None of the potential risk factors (sex, age, site, SUV_max_, SUV_mean_, MTV, TLG) for recurrence and metastasis in high grade PGISL was identified as a risk factor on univariate and multivariate Cox regression analysis.

**Conclusion:**

Metabolic tumor parameters are not predictive markers in high-grade PGISL, especially in diffuse large B cell variant and primary gastric lymphoma. The first implications suggest they will not play a role in patient management.

## Abstract (tr)

**Amaç:**

Primer lenfatik alanlar dışındaki dokulardan kaynaklanan Non-Hodgkin lenfomalara primer ekstranodal lenfoma (PEL) denmektedir. Gastrointestinal sistemin PEL’i (PGISL) buradaki lenfatik dokulardan köken alır. ^18^F-FDG PET/BT lenfomalarda genel ve hastalıksız sağkalım açısından yüksek prognostik değere sahiptir. Amacımız düşük grad ve yüksek grad PGISL’de primer evreleme ^18^F-FDG PET/BT’de tutulum şekillerini, özelliklerini, yüksek grad PGISL’de metabolik tümör parametrelerinin prognostik önemi ile birlikte araştırmaktır.

**Yöntem:**

2004-2015 yılları arasında PGISL (evre 1-2) tanısı konmuş 39 hasta bu retrospektif kohort çalışmaya dahil edildi. Hastalara tedaviden önce primer evreleme ^18^F-FDG PET/BT çekilmiş ve maksimum standardize uptake değeri (SUV_maks_), ortalama standardize uptake değeri (SUV_ortalam_a), metabolik tümör hacmi (MTV) ve total lezyon glikolizi (TLG) gibi metabolik tümör parametreleri hesaplanmıştı. Düşük grad ve yüksek grad PGISL metabolik tümör parametreleri açısından karşılaştırıldı. Yüksek grad PGISL’de Cox regresyon modelleri üzerinden hastalıksız sağkalım ile ilişkili faktörler tespit edildi.

**Bulgular:**

Düşük grad ve yüksek grad PGISL arasında SUV_maks_, SUV_ortalama_, MTV, TLG, nüks, mortalite, genel ve hastalıksız sağkalım açısından istatistiksel olarak anlamlı fark saptanmıştır. Yüksek grad PGISL’de nüks ve metastaza etki eden tüm potansiyel risk faktörlerinin (cinsiyet, yaş, site, SUV_maks_, SUV_ortalama_, MTV, TLG) tek ve çok değişkenli Cox regresyon analizinden sonra metabolik tümör parametrelerinin bir risk faktörü olmadığı görülmüştür.

**Sonuç:**

Metabolik tümör parametreleri özellikle diffüz büyük B hücreli varyant ve primer gastrik lenfoma başta olmak üzere yüksek grade PGISL’nin prognoz tahmininde faydalı değildir. İlk izlenimler hasta yönetiminde bir rolleri olamayacağı yönündedir.

## INTRODUCTION

Non-Hodgkin’s lymphomas (NHLs) arising from tissues other than primary lymphatic sites [lymph nodes (LNs), bone marrow, spleen, thymus and Waldeyer’s ring of pharyngeal lymphatics] are classified as primary extranodal lymphoma (PEL) (1). Although PEL can be seen in almost any site, gastrointestinal system (GIS) is the most frequent site (2). Thus, PEL of the GIS (PGISL) is a NHL originating from lymphatic tissues of the gastrointestinal tract. The most commonly affected site is the stomach (50-60%) followed by the small intestine (approximately 30%), colon, very rarely pancreas and the liver (3). Approximately 1/2 to 2/3 of GIS NHLs are diffuse large B-cell (DLBC) lymphomas (1).

Primary gastric lymphoma (PGL) constitutes less than 5% of all gastric neoplasms (4). It is the most common site in all PEL patients with an incidence of 4-20%, and a preponderance in men over the age of 50 (5). Histologically, PGL is predominantly high-grade, DLBC or low-grade mucosa-associated lymphoid tissue (MALT), also defined as extranodal marginal zone B cell lymphoma (6). MALT lymphoma is the most common variety in PGL (7). A heterogeneous group of lymphomas including MALT, DLBC, mantle cell (MC), Burkitt and T-cell affect the small bowel (7). Primary colon lymphoma has features similar to small bowel disease with wall thickening without obstruction (8). 30-50% of patients with small bowel lymphoma initially present with an abdominal emergency (9).

The treatment of GIS lymphomas is controversial and depends on histologic type and disease stage (10). Although computed tomography (CT), 18-fluorodeoxyglucose positron emission tomography (^18^F-FDG PET) and ^18^F-FDG PET/CT are used to stage PEL, CT is the most commonly used imaging modality for the management of patients with lymphomas (2). Most patients present with residual masses after treatment (11). A decrease in the size of a lymphomatous mass after treatment is considered as treatment response. However, a decrease in size on CT does not occur in case of fibrosis, necrosis and inflammation (11). Therefore, CT cannot differentiate residual disease from fibrosis.

^18^F-FDG PET is a superior imaging technique that proved its utility especially in Oncology. It can display functional alterations that precede anatomical changes. Several limitations of CT in lymphomas can be overcome with ^18^F-FDG PET. Besides, integration of CT to ^18^F-FDG PET creates a superior imaging modality combining anatomical detail with functional information, which results in excellent accuracy and detection capability. With all these advantages, ^18^F-FDG PET/CT is being widely used in the primary staging, evaluation of treatment response, and restaging of PGISL just like other types of HL and many NHL lymphomas.

^18^F-FDG PET/CT also has a high prognostic value with respect to overall survival (OS) and disease-free survival (DFS). The semi-quantitative measurement of standardized uptake value (SUV) is an easy-to-calculate and noninvasive index reflecting ^18^F-FDG metabolic rate. Its assessment has additional prognostic value in early response to treatment and long-term outcome in lymphoma patients and improves the prognostic value of the test manifestly as compared to visual analysis (12). Many studies have proven the effectiveness of ^18^F-FDG PET/CT for primary staging, restaging and evaluation of treatment response in lymphomas (13). Despite the high incidence of PEL of the GIS, only a few studies with limited number of patients have been published in the literature on the use of ^18^F-FDG PET/CT in the management of these patients (14). Moreover, to the best of our knowledge no prior studies have been published on metabolic tumor parameters in this selective subgroup of lymphoma. This study was conducted to investigate the usefulness of metabolic tumor indices on primary staging ^18^F-FDG PET/CT for prognosis estimation in primary extranodal high-grade GIS lymphomas. We also studied the uptake patterns and properties of metabolic tumor parameters in different histological subtypes of PGISL.

## MATERIALS AND METHODS

Thirty-nine patients with PGISL (only stage 1-2 disease) from 2004 to 2015 were enrolled in this retrospective cohort study. The cases were histopathologically proven by excisional biopsy. The study was conducted at the Nuclear Medicine Department of a training and research hospital of a medical school. Primary staging ^18^F-FDG PET or ^18^F-FDG PET/CT have been performed for all patients prior to treatment. Disseminated nodal disease secondarily involving GIS and Hodgkin lymphoma (HL) with GIS involvement were excluded. Patients who didn’t have primary staging ^18^F-FDG PET/CT and inadequate follow-up were also not included. Involvement of GIS as the predominant site with a few minor draining LNs only were also categorized as PGISL and included in the study. These patients were treated and followed up by the Medical Oncology Department of our hospital. CD20 (+) cases were treated by R-CHOP protocol (rituximab, cyclophosphamide, doxorubicin, vincristine, prednisolone), while CD20 (-) cases received CHOP. The patients were followed by clinical history, physical examination, lactate dehydrogenase and sedimentation rate measurement, complete blood count, liver function tests, CT and/or ^18^F-FDG PET/CT. Information and data were obtained from clinic follow-up files, radiation therapy records, physician records of other departments at our hospital or personal contact with the patients on telephone.

### ^18^F-FDG PET/CT Imaging Protocol

Patients fasted for 6 hours and their blood glucose level had to be under 150 mg/dL before the injection of an activity of 370-555 MBq of ^18^F-FDG according to patient’s weight. Image acquisitions were performed 1 hour later with an integrated PET/CT scanner (Discovery 690-GE Healthcare). Unenhanced low dose CT and PET emission data were acquired from mid-thigh to the vertex of the skull in supine position with the arms raised over head. CT data was obtained by automated dose modulation of 120 kVp (maximal 100 mA), collimation of 64×0.625 mm, measured field of view (FOV) of 50 cm, noise index of 20% and reconstructed to images of 0.625 mm transverse pixel size and 3.75 mm slice thickness. PET data was acquired in 3D mode with scan duration of 2 min per bed position and an axial FOV of 153 mm. The emission data was corrected in a standardized way (random, scatter and attenuation) and iteratively reconstructed (matrix size 256×256, Fourier rebinning, VUE Point FX [3D] with 3 iterations, 18 subsets).

### Visual and Quantitative Interpretation

Quantitative PET/CT parameters used in the study include maximum standardized uptake value (SUV_max_), average standardized uptake value (SUV_mean_), metabolic tumor volume (MTV) and total lesion glycolysis (TLG). They were calculated according to a standard protocol on a dedicated workstation (Volumetrix for PET/CT and AW volume share 4.5, GE Healthcare, Waukesha, WI, USA). SUV_max_ and SUV_mean_ corrected for body weight were computed by standard methods from the activity at the most intense voxel in three-dimensional tumor region from the transaxial whole body images on attenuation-corrected PET/CT images. MTV (cm^3^) was measured with semiautomatic PET analysis software using an automatic isocontour threshold method based on a theory of being greater than 42% of the SUV_max_ value within the tumor. TLG values were calculated by multiplying MTV and SUV_mean_. The corresponding CT scan of lesions were used as a guideline to demarcate them if their boundaries were difficult to define for the calculation of SUV_max_ (15).

We retrospectively examined demographic characteristics, clinical findings, histology, clinical stage, response to treatment and outcome of the patients. OS was defined as the interval between diagnosis and death of any cause including ones other than the disease itself or until last follow-up. DFS was defined as the interval between diagnosis to detection of relapse or until last follow-up. Informed consent was waived due to the retrospective design of the study using records, documents and data of patients referred to our clinic for the test. Ann-Arbor staging system and definitions were used in this study. The study was approved by The study was approved by a Gülhane Training and Research Hospital Local Ethics Committee (Date: 17.02.2016, Protocol number: 40).

### Statistical Analysis

The data were analyzed by IBM Corp. Released 2013. IBM SPSS Statistics for Windows, Version 22.0. Armonk, NY:IBM Corp. Number and percentage values were used for description of categorical data; while mean, median, standard deviation, minimum (min) and maximum (max) values were used for description of continuous data. Fisher’s exact test was used for comparison of high-grade and low-grade PGISL in terms of sex, recurrence and mortality; the chi-square test for site; the Student-t test for age and SUV_max_; the Mann Whitney-U test for SUV_mean_, MTV, TLG, OS and DFS. Univariate Cox regression analysis was used to determine factors that correlated with DFS and OS of high-grade PGISL DLBC variant. Univariate and multivariate analysis (Cox regression, Forward LR models) were performed for the evaluation of factors impacting recurrence. The variables having a value of p<0.5 were further included into multivariate analysis. ROC curve was drawn to evaluate the diagnostic value of SUV_max_, SUV_mean_, MTV and TLG. They were dichotomized by splitting into two groups according to ROC curve. Kaplan-Meier method with log-rank test was used to compare DFS times of metabolic tumor parameters. The study was approved by our Institutional Review Board.

## RESULTS

**Figure 1:**
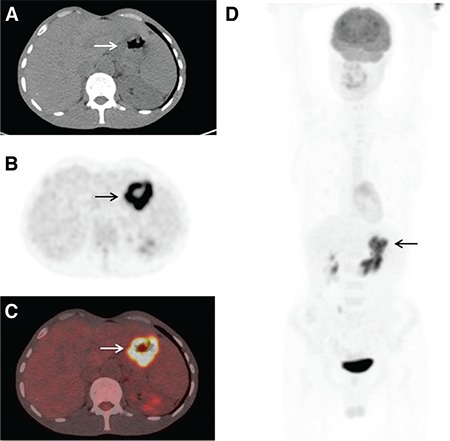
The SUV_max_, SUV_mean_, metabolic tumor volume and total lesion glycolysis values of a 58-year old male patient with primary gastric lymphoma diffuse large B-cell variant were 11.5, 5.2, 35 cm^3^ and 176, respectively, on trans-axial computed tomography (A), positron emission tomography (B), fusion (C) and maximum intensity projection (D) images of baseline 18-fluorodeoxyglucose positron emission tomography (arrows). He responded to treatment and his disease-free survival and overall survival are 92 months

There were 23 DLBC lymphomas (59%), 12 MALT lymphomas (31%), 2 T-cell lymphomas (5%), one MC lymphoma and one Burkitt lymphoma in the study. Totally, we had 26 high-grade PGISL (67%) and 13 low-grade PGISL (33%). Twenty-eight of the patients were male (72%) and 11 were female (28%). Mean age of the patients was 57±15 years (21-87 years). The site distribution amongst these 39 patients was as follows: 23 PGL (59%) (Figure 1), 7 small bowel (18%), 7 primary colon lymphoma (18%), one primary pancreas lymphoma, and one primary liver lymphoma. Thirty-four out of 39 (87%) cases had stage 1, while 5 (13%) had stage 2 disease. Patient characteristics and demographic findings, clinic-pathologic features and follow-up data of high-grade and low-grade PGISL are presented in Table 1 and Table 2. There were statistically significant differences between high-grade and low-grade PGISL in terms of SUV_max_, SUV_mean_, MTV, TLG, recurrence, mortality, DFS and OS. The comparison of high-grade and low-grade PGISL features is shown in Table 3.

Univariate Cox regression was performed for all potential risk factors (sex, age, site, SUV_max_, SUV_mean_, MTV, TLG) impacting recurrence and/or metastasis (met/rec). Site, SUV_max_, SUV_mean_, was determined as statistically significant on univariate analysis. The results of univariate Cox regression analysis are shown in Table 4. Factors with p<0.5 on univariate analysis (sex, age, SUV_max_, SUV_mean_, and site) were evaluated further with multivariate model. Only sex remained statistically significant on multivariate analysis (p=0.037).

**Figure 2:**
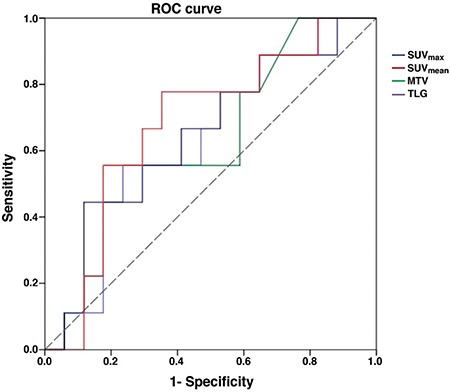
ROC curve of SUV_max_, SUV_mean_, metabolic tumor volume and total lesion glycolysis for high-grade primary extranodal lymphomas of the gastrointestinal system MTV: Metabolic tumor volume, TLG: Total lesion glycolysis

**Figure 3:**
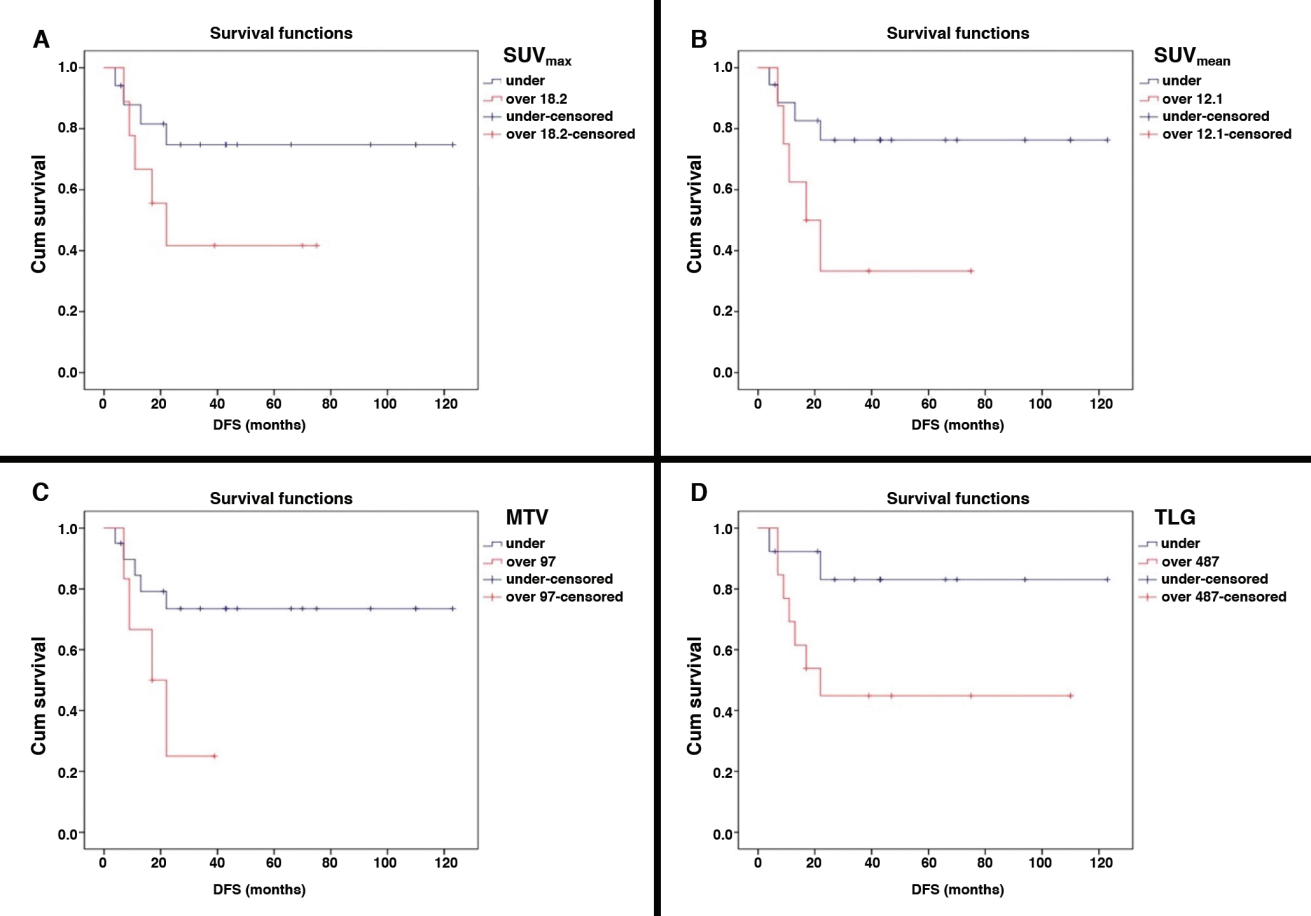
Survival graphic of high-grade primary extranodal lymphomas of the gastrointestinal system diffuse large B-cell variant obtained by univariate Cox regression test. Kaplan-Meier curves of SUV_max_ with a cut-off value of 18.2 (A), SUV_mean_ with a cut-off value of 12.1 (B), metabolic tumor volume with a cut-off value of 97 cm^3^ (C), total lesion glycolysis with a cut-off value of 487 (D) for high-grade primary extranodal lymphomas of the gastrointestinal system MTV: Metabolic tumor volume, TLG: Total lesion glycolysis, DFS: Disease-free survival

Within the group of patients with high-grade PGISL, 8 patients (31%) died and 9 patients (34.5%) developed met/rec during follow-up. Four patients died of causes other than the disease (cardiovascular events, cerebrovascular diseases, aging, etc). Four patients died of disease related reasons (extensive metastasis and related complications). OS at the 5^th^ year was 77%, and 73% at the 10^th^ year. The average time to detection of met/rec was 12.6 months (4-22). DFS was 80.5% and 65% at the first and second years, respectively. ROC curve was drawn to evaluate the diagnostic value of SUV_max_, SUV_mean_, MTV and TLG in high-grade PGISL (Figure 2). Survival graphics of high-grade PGISL-DLBC variant obtained by univariate Cox regression analysis was plotted. Metabolic tumor parameters were dichotomized by splitting into two groups according to ROC curve. Kaplan-Meier method with log-rank test was used to compare DFS and SUV_max_, SUV_mean_, MTV and TLG. Kaplan-Meier curves of SUV_max_ (A), SUV_mean_, (B), MTV (C), and TLG (D) with their cut-off values are illustrated in Figure 3. The sensitivity and specificity rates of SUV_max_ SUV_mean_, MTV, and TLG according to cut-off values for high-grade PGISL are represented in Table 5.

## DISCUSSION

^18^F-FDG PET/CT was performed in 447 patients with NHL during the study period in our department. The incidence of PGISL in our study group is 8.7% (39/447), evidently under the rates reported in the literature due to our selective population. The peak incidence is in the 6^th^-7^th^ decades with a male predominance (16). The mean age of our group was 57 years with a male preponderance, in line with the literature. The risk of NHL is higher in men than in women, but there are certain types of NHL that are more common in women (16). However, there are no studies that have identified sex as a risk factor in the literature. We found that sex was the only factor affecting recurrence on multivariate analysis. Although female sex was the only risk factor for recurrence in our study, we attribute this finding to the insufficient sampling number, and thus believe this finding might not be clinically important.

Gastrointestinal tract is the most common extranodal site of NHL (17). The stomach is the most frequently involved (60-74% of cases) site, followed by the duodenum and small bowel (10-20%), ileocecal region (7-10%), and colon (<10%) (18). Our results are consistent with these reports, except that the colon was affected in a greater percentage of patients (equal to small intestine involvement) than previous studies. The stomach is the most common site of primary GIS lymphoma, and gastric MALT lymphoma is the most common type (7). In our study, 39% of gastric lymphomas were MALT type while 61% were DLBC variant. Our incidence of gastric DLBC was markedly higher than gastric MALToma. This result is in contrast to the literature. MALT, DLBC, MC, Burkitt and T cell can all be seen in the small bowel and colon. The most common variant was DLBC (59%) in our patients followed by MALT (31%). These findings are also completely in agreement with previous reports.

Primary hepatic lymphoma (PHL) is very rare and up to date, only about 300 cases were published in the literature. Of all PELs, only 0.4% occurs in the liver (9). The most common variant in the liver is DLBC, accounting in one study for 71% of all cases (19). Our sole PHL patient was a DLBC subtype with poor prognosis (recurred at the 13^th^, died at the 21^st^ month). Primary pancreatic lymphoma (PPL) is defined as an extranodal lymphoma arising in the pancreas with the bulk of the tumor localized to the pancreas. PPL is a very rare disease, accounting for less than 0.5% of pancreatic tumors (20). The only case with PPL in our study was a DLBC variant and his clinical course was fatal with high metabolic parameters (recurred at the 9^th^, died at the 17^th^ month).

There is a correlation between ^18^F-FDG uptake and histologic grade of lymphoma, since rapidly proliferating lymphoma cells have a high metabolic rate and aggressive subtypes of NHL take up high levels of ^18^F-FDG (21). The ^18^F-FDG PET imaging in our study showed that high-grade PGISL had high ^18^F-FDG activity as confirmed with high metabolic tumor parameters. Particularly, DLBC variants exhibited usually high ^18^F-FDG accumulation. Although low-grade PGISL lesions were identified with difficulty especially in MALT lymphoma, MALT types had variable (usually moderate) uptake in this study. Jerusalem et al. (22) reported that low-grade NHLs such as follicular lymphoma and MC lymphoma do not demonstrate ^18^F-FDG-avidity to the same degree with high-grade lymphomas, but they are still ^18^F-FDG-avid enough to be detected. There was a significant difference in terms of SUV_max_, SUV_mean_, MTV and TLG between high-grade and low-grade PGISL in our patients (p=0.038, p=0.024, p<0.001, p<0.001, respectively).

The majority of patients with MC present with advanced-stage disease and often have extranodal sites. These patients have a poor prognosis with a median survival of 3 to 4 years (23). One patient with MC lymphoma in this study accumulated mild ^18^F-FDG and had a good prognosis, nevertheless, our patient had stage 1 disease. MALT lymphoma is the third most common NHL following DLBC and follicular lymphoma (24). Most studies report that MALT lymphomas demonstrate moderate to high ^18^F-FDG accumulation while a few studies with limited number of patients claim that ^18^F-FDG PET imaging is unreliable in case of primary extranodal MALT lymphoma (25). We detected usually moderate uptake and no recurrence or death in our cases with MALT lymphoma. Burkitt lymphoma is a highly aggressive B-cell NHL. There was only one patient with Burkitt type among our cases. The ^18^F-FDG uptake was high in that patient with increased metabolic tumor parameters, and the tumor recurred at 7 months. T-cell lymphomas are generally aggressive neoplasms. Two of our cases with T-cell variant had a favorable prognosis with moderate ^18^F-FDG uptake. Six gastric DLBC, one PHL and one PPL were responsible for deaths. We observed complete remission in 26 patients (13/26 in the high-grade group, all patients in the low-grade group). In our study group, DFS was identified as 77% and OS as 79.5% with a mean follow-up of 65 months (6-161). These results are in agreement with other studies in the literature.

Quantitative metabolic parameters (SUV_max_, SUV_mean_, MTV, TLG) obtained from initial staging PET/CT has been used in prognosis estimation and evaluation of treatment response for many cancers and lymphomas. Tumor cells utilize glucose at a higher metabolic rate as displayed by abnormal ^18^F-FDG uptake. SUV measures this consumption rate that correlates with cellular metabolism (26). SUV_max_ is the first one of metabolic tumor parameters, which represents the highest ^18^F-FDG uptake within the tumor volume. SUV_mean_ represents the average activity in a tumor burden. Recently, volume-based metabolic parameters (MTV and TLG) have been used to detect recurrent disease to the best of our knowledge, the literature lacks any study pertaining to the use of these metabolic factors for prognosis estimation in high-grade PGISL.

Although a study specifically on this subject has not been conducted, similar studies with SUV_mean_ has been previously reported. Most studies investigated metabolic tumor parameters for different sites or unique variants and aimed to evaluate them in treatment. Esfahani et al. (27) stated TLG as the most significant parameter for predicting detection of recurrence in DLBC on initial and interim PET. Gallicchio et al. (28) reported that quantitative parameters were helpful in the management of patients with DLBC. Recently, TLG emerged as a remarkable predictor in many cancers and lymphomas, since it contributes to patient management by assessing both tumor volume and metabolism. Ceriani et al. (29) suggested TLG as the most powerful predictor on baseline PET/CT in DLBC. However, there are no studies on these parameters in PGISL. To the best of our knowledge, our study is the first one in which the prognosis of high-grade PGISL was predicted by these metabolic indicators.

Previously reported studies on PGISL are usually focused on evaluating response to treatment by SUV_max_ alone. Phongkitkarun et al. (15) reported that SUV_max_ plays an important role in the evaluation of treatment response. Kumar et al. (2) identified high SUV_max_ as a strong predictor of recurrence after completion of therapy. We aimed to investigate the efficacy of all metabolic tumor parameters for predicting prognosis in high-grade PGISL. After evaluation of all potential risk factors effecting met/rec with univariate Cox regression analysis and multivariate model, none of the study parameters were detected to have a statistically significant correlation with DFS in our study. Analysis of the diagnostic value of these parameters by ROC curve determined high sensitivity rates for SUV_max_, SUV_mean_, MTV and a high specificity rate for TLG with the selected cut-off values (487 for TLG). The results indicate that high-grade PGISL, especially DLBC subtype and PGL, tend to have high metabolic parameters indirectly reflecting high mitotic activity of tumor cells. This particularity is perhaps responsible for responding well to treatment. In other words, first results indicate that metabolic tumor parameters are not prognostic in high-grade PGISL despite their high values.

## CONCLUSION

Metabolic tumor parameters are not predictive markers in primary high-grade gastrointestinal lymphomas, especially in DLBC variant and PGL. The findings suggest they will not play a role in patient management. Female sex was identified as the single risk factor for recurrence.

## Figures and Tables

**Table 1:**
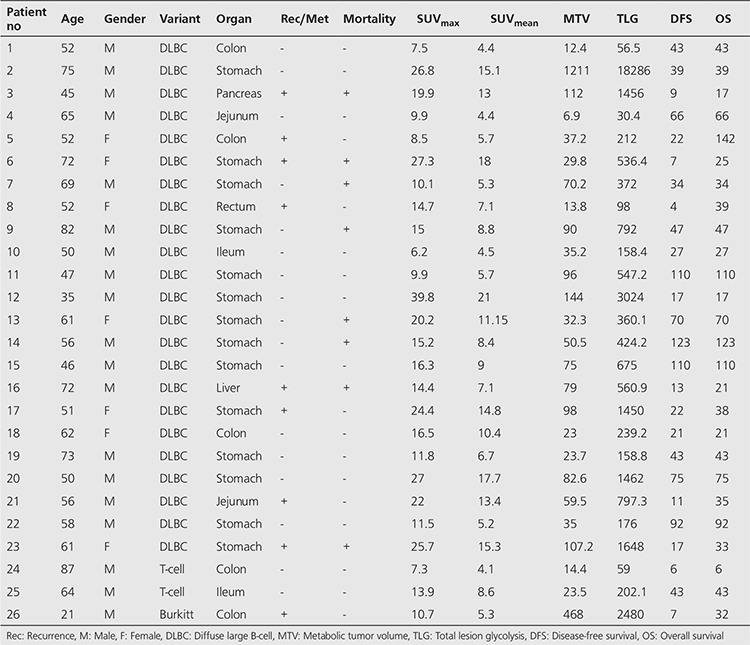
Demographic characteristics of patients with high-grade primary extranodal lymphomas of the gastrointestinal system, clinic-pathologic features and follow-up data

**Table 2:**
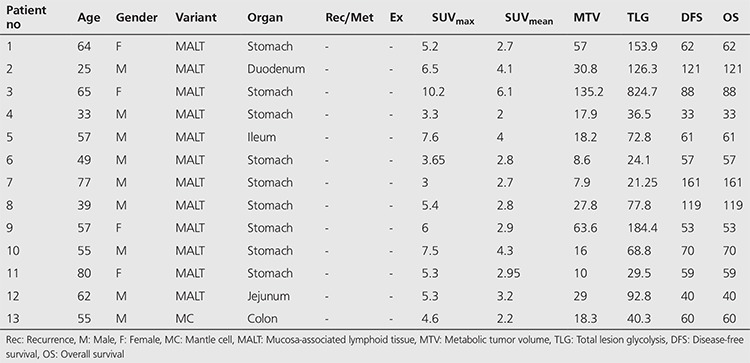
Demographic characteristics of patients with low-grade primary extranodal lymphomas of the gastrointestinal system, clinic-pathologic features and follow-up data

**Table 3:**
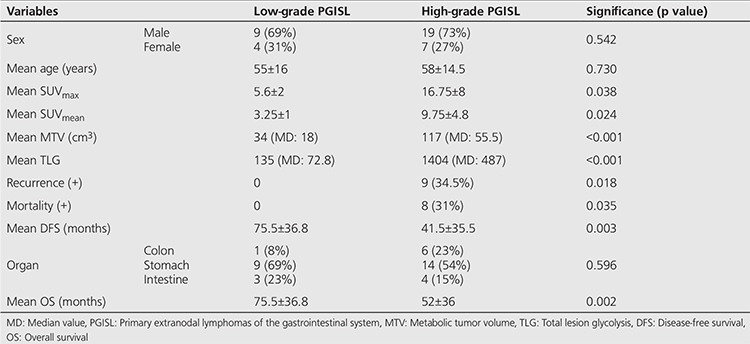
Comparison of high-grade and low-grade primary extranodal lymphomas of the gastrointestinal system in terms of patient characteristics, follow-up data and metabolic tumor parameters

**Table 4:**
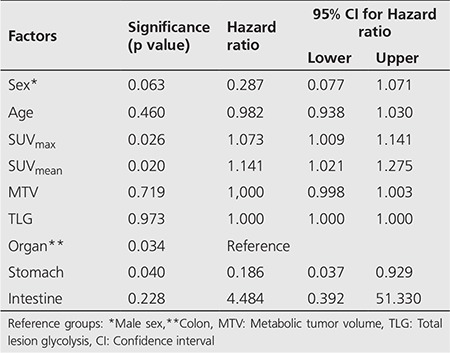
Univariate Cox regression analysis of high-grade primary extranodal lymphomas of the gastrointestinal system diffuse large B-cell variant

**Table 5:**
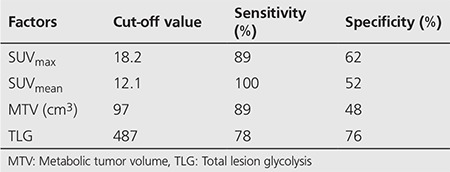
Cut-off value, sensitivity and specificity rates of SUV_max_, SUV_mean_, metabolic tumor volume and total lesion glycolysis in high-grade primary extranodal lymphomas of the gastrointestinal system
